# Over-toasting dehulled rapeseed meal and soybean meal, but not sunflower seed meal, increases prececal nitrogen and amino acid digesta flows in broilers

**DOI:** 10.1016/j.psj.2022.101910

**Published:** 2022-04-08

**Authors:** Miranda L. Elling-Staats, Arie K. Kies, Myrthe S. Gilbert, René P. Kwakkel

**Affiliations:** ⁎ Animal Nutrition Group, Department of Animal Science, Wageningen University, The Netherlands; † DSM Nutritional Products, Animal Nutrition and Health - EMEA, Kaiseraugst, Switzerland

**Keywords:** broiler, protein, cecal fermentation, amino acid digestibility, prececal digesta flow

## Abstract

Poorly digestible proteins may lead to increased protein fermentation in the ceca of broilers and hence, the production of potentially harmful metabolites. To evaluate effects of protein fermentation on gut health, an experimental contrast in ileal nitrogen (**N**) and amino acid (**AA**) flow is required. Therefore, our objective was to develop a model that creates a contrast in protein fermentation by increasing the prececal flow of protein within ingredients. To this end, we used additional toasting of protein sources and evaluated the effect on prececal N and AA flows. One-day-old Ross 308 male broilers (n = 480) were divided over 6 dietary treatments, with 8 replicate pens with 10 broilers each. Diets contained 20% of a regular soybean meal (**SBM**), high protein sunflower seed meal (**SFM**) or a dehulled rapeseed meal (**dRSM**) as is, or heat damaged by secondary toasting at 136°C for 20 min (**tSBM, tSFM**, or **tdRSM**). Ileal and total tract digesta flows of N and AA were determined with 5 birds per pen in their third week of life using an inert marker (TiO_2_) in the feed. Additional toasting increased the feed conversion ratio (**FCR**) only in birds fed dRSM (1.39 vs. 1.31), but not SBM and SFM (interaction *P* = 0.047). In SBM, additional toasting increased the flow of histidine, lysine, and aspartate through the distal ileum and excreted, while in SFM it had no effect on flows of N and AA. Toasting dRSM increased the prececal flows and excretion of N (862 vs 665 and 999 vs 761 mg/d, respectively) and of the AA. Of the ingredients tested, toasting dRSM is a suitable model to increase protein flows into the hind-gut, permitting the assessment of effects of protein fermentation.

## INTRODUCTION

Highly digestible protein sources are expected to become less available for animal production due to a growing world population (Boland et al., 2013). This will increase the inclusion of, generally less digestible, byproducts into animal feed. In broilers, pre-cecal protein digestion of soybean meal (**SBM**), which is commonly used, is 90%. Alternative protein sources have a lower digestibility, for example, 84% for sunflower seed meal (**SFM**) and 76% for rapeseed meal (**RSM**; Lemme et al., 2004).

Protein that remains undigested and unabsorbed in the small intestine will move towards the ceca and large intestine and may become available for fermentation by microbes. A high level of poorly digestible crude protein (**CP**) in the diet of broilers reduces growth and productivity (De Lange et al., 2003; Bryan et al., 2019) and could increase the susceptibility to diseases, such as necrotic enteritis and coccidiosis (Sharma et al., 1973; Drew et al., 2004; Fernando et al., 2011). This is likely the result of protein fermentation (**PF**) in the ceca of these birds. Metabolites produced during PF, such as ammonia, hydrogen sulfide, phenols, indoles, and biogenic amines, are potentially toxic and may result in (gut) health issues, as reviewed by Qaisrani et al. (2015) and Gilbert et al. (2018). Studies in mammals have associated PF metabolites in the hind-gut, resulting from high CP intake, with a number of gut health effects. Examples of such effects are: 1) poor fecal consistency and high fecal moisture content (Nery et al., 2012), 2) increased expression of proinflammatory cytokines in the colon (Pieper et al., 2012; Villodre Tudela et al., 2015), 3) reduced expression of tight junction proteins in the colon (Richter et al., 2014), 4) reduction in the colonic expression of monocarboxylate transporter 1, an important transporter of butyrate (Villodre Tudela et al., 2015), and 5) increased expression of mucus production genes (Pieper et al., 2012; Lan et al., 2015). In chicken, however, little is known about the effects of PF metabolites on gut health.

To study this, a dietary model which creates a contrast in cecal PF is required. This contrast should not contain confounding effects of other nutrients, specifically carbohydrates, as many bacteria appear to prefer using carbohydrates as an energy source over protein (Jeaurond et al., 2008; Rehman et al., 2008). Therefore, replacing a highly digestible protein source, such as SBM, with a poorly digestible protein source, such as RSM with a higher fiber content, is not a suitable model, as it did not result in an increase in cecal concentrations of PF metabolites (Qaisrani et al., 2014), as likely both the flow of undigested protein as carbohydrates towards the ceca increased.

A possible solution to this confounding effects of other nutrients is a within protein ingredient contrast created by hydrothermal processing (e.g., toasting). Toasting is used in the industry to reduce the concentration of antinutritional factors and to improve digestibility of proteins in ingredients, such as SBM. However, severe toasting may reduce ileal digestibility of dietary proteins, due to their aggregation (Salazar-Villanea et al., 2016a, 2016b, 2018). The flow of protein into the hind-gut is then, consequently, higher.

This study aimed to evaluate the effect of additional severe toasting of three protein sources on the protein and amino acid digesta flows towards the ceca in order to determine which contrast would be most suitable to study effects of cecal PF on broiler (gut) health. For this purpose we used soybean meal, high-protein sunflower seed meal, and dehulled rapeseed meal. These meals were fed either as commonly processed or after receiving additional toasting to reduce protein digestibility.

## MATERIAL AND METHODS

### Ethics Statement

A project license was granted by the Central Committee for Animal Experimentation (The Hague, the Netherlands) after approval by the Animal Care and Use Committee of Wageningen University and Research (AVD1040020171667, Wageningen, The Netherlands). The experiment was approved by the Animal Welfare Body of Wageningen University and Research (2017.W-0025.002).

### Experimental Design

Six diets were designed to evaluate the effects of additional toasting of three protein sources. The sources used were soybean meal, high-protein sunflower seed meal, and dehulled rapeseed meal. These sources were included into the diet as-is (**SBM, SFM**, or **dRSM**, respectively), or after an extra heat treatment (**tSBM, tSFM, tdRSM**, respectively).

### Birds and Housing

A total of 480 one-day old ROSS 308 male broilers were obtained from a commercial hatchery (Kuikenbroederij Morren B.V., Lunteren, the Netherlands). Upon arrival, birds were wing-tagged, weighed and randomly assigned to one of 48 pens divided over two rooms and organized into 4 blocks per room. Experimental diets were provided in 8 replicate pens in a randomized order within block, with 10 birds in each pen. Pens were 2 m^2^ and the floor was slatted. Cardboards with a thick layer of pellets of finely ground lignocellulose (SoftCell) were placed on the slatted floors. Each pen contained a large round feeder, three drinking nipples and a perch. Birds had ad libitum access to feed and water. Temperature was maintained at 32°C to 34°C during the first 3 d and was gradually decreased to 20°C to 22°C on d 30. Relative humidity in the rooms was set at 40% (range: 20–50%). The lights were kept on for 23 h during the day of arrival, after which the dark period was increased with one hour every day up to 8 h.

Sex errors were noticed in the second week of the experiment. Nineteen percent of the broilers appeared to be female. On d 14, broilers were rearranged within their experimental treatment to create as similar as possible numbers of females per pen.

### Diets

The SBM and SFM were obtained via ABZ-Diervoeding (Nijkerk, the Netherlands) and originated from Brazil and Germany, respectively. The dRSM was produced in the pilot plant of Olead (Pessac, France). Full fat rapeseed was dehulled with a Ripple flow impact dehuller (CPM), and the kernels were subsequently pressed with a MBU20 Press (OLEXA, Arras, France).

The heat-damaged ingredients, tSBM, tSFM, and tdRSM, were created by pressurized steam toasting of SBM, SFM, and dRSM, respectively. Toasting was done using the lab scale steam toaster of Wageningen University, Netherlands. During steam toasting, the machine was pressurized up to 40 kPa, allowing it to reach the maximum temperature of 136°C. This temperature was then maintained for 20 min. After toasting, moist meals were dried in an oven at 70°C overnight. The diets were mixed and pelleted at the pilot plant of ABZ-Diervoeding in Leusden, The Netherlands. Test ingredients were included into the diet at 20%. Experimental diets consisted of a starter diet, which was fed from hatch to d 14, and a grower diet fed from d 14 onward. All diets were pelleted at 65°C. Titanium dioxide (TiO_2_, 1.0 g/kg diet) was included as an indigestible marker at the expense of maize, in the diets fed from d 21 onward. Diets were formulated to meet or exceed broiler requirements. Diet compositions of the starter and grower diets are shown in Tables 1 and 2, respectively. The diet formulations within an ingredient group (e.g., SBM and tSBM) were kept similar. Hence, no corrections were made for the heat damage of nutrients in the diets including the extra toasted test ingredients. The analyzed AA composition of the grower diets is shown in Table 3.

**Table 1:** Ingredient and nutrient composition of experimental starter diets (fed from 0 to 14 d of age) containing soybean meal (SBM), sunflower seed meal (SFM) and dehulled rapeseed meal (dRSM) as-is or after additional toasting (tSBM, tSFM, tdRSM, respectively).

	SBM	tSBM	SFM	tSFM	dRSM	tdRSM
Ingredients, g/kg as-fed basis						
Soybean meal	200.0	-	-	-	-	-
Toasted Soybean meal	-	200.0	-	-	-	-
High protein Sunflower seed meal	-	-	200.0	-	-	-
Toasted HP Sunflower seed meal	-	-	-	200.0	-	-
Dehulled Rapeseed meal	-	-	-	-	200.0	-
Toasted dehulled Rapeseed meal	-	-	-	-	-	200.0
Wheat	200.0	200.0	112.4	112.4	103.4	103.4
Maize	322.7	322.7	398.0	398.0	398.0	398.0
Maize gluten (Prairy gold)	10.0	10.0	10.8	10.8	27.7	27.7
Finely ground oat hulls	30.0	30.0	-	-	-	-
Soy protein (Provisoy)	70.0	70.0	60.3	60.3	68.9	68.9
Peas	74.2	74.2	100.0	100.0	99.0	99.0
Potato protein	9.4	9.4	30.0	30.0	27.0	27.0
Soybean oil	28.5	28.5	33.9	33.9	27.6	27.6
Chicken fat	7.5	7.5	7.5	7.5	7.5	7.5
Limestone	12.5	12.5	12.4	12.4	10.4	10.4
Mono calcium phosphate	10.7	10.7	10.0	10.0	9.8	9.8
Sodium bicarbonate	3.9	3.9	4.7	4.7	3.6	3.6
Salt	0.7	0.7	-	-	0.7	0.7
DL-Methionine	2.7	2.7	1.9	1.9	2.0	2.0
L-Valine	3.6	3.6	-	-	-	-
L-Tryptophan	-	-	2.2	2.2	0.2	0.2
L-lysine HCl	2.5	2.5	5.0	5.0	3.5	3.5
L-Threonine	1.0	1.0	1.0	1.0	0.7	0.7
Phytase premix^1^	4.0	4.0	4.0	4.0	4.0	4.0
Premix^2^	6.0	6.0	6.0	6.0	6.0	6.0
Analyzed nutrients, g/kg DM (unless otherwise stated)						
DM, g/kg	900.1	897.4	887.6	897.2	901.4	854.7
Crude protein	250.7	248.8	252.6	249.0	255.6	265.2
Crude fat	62.2	58.6	58.9	85.7	77.0	19.5
Starch	391.5	401.8	420.7	405.9	391.7	416.3
Crude fiber	44.2	44.1	41.6	40.3	40.0	44.0
Soluble NSP (calculated)	30.0	30.0	27.8	27.8	29.8	29.8
Insoluble NSP (calculated)	119.5	119.5	123.6	123.6	120.8	120.8
Metabolizable energy (MJ, calculated)	12.0	12.0	12.0	12.0	12.0	12.0

1 The phytase premix supplied 1000 FTU/kg (calculated).

2 Premix provided per kilogram of complete diet: 12000 IU vitamin A, 4000 IU vitamin D3, 60 mg vitamin E, 3 mg vitamin K3, 3 mg vitamin B1, 9 mg vitamin B2, 60 mg vitamin B3, 18 mg pantothenic acid, 6 mg vitamin B6, 30 µg B12, 300 µg biotin, 600 mg choline chloride, 1.8 mg folic acid, 35 mg linoleic acid (C18:2), 120 mg Ca, 12.2 mg P, 8.2 mg Mg, 17.8 mg K, 0.7 mg Na, 133.6 mg Cl, 91.8 mg S, 68.6 mg Fe (as FeSO4), 2.4 mg I (as Ca(IO3)2), 15.0 mg Cu (as CuSO4), 90.5 mg Mn (as MnO), 84.1 mg Zn (as ZnSO4), 0.3 mg Se (as Na2SeO3), 1 mg butylated hydroxytoluene, 1 mg propyl gallate, 2.4 mg citric acid. Ground wheat was used as carrier.

**Table 2:** Ingredient and nutrient composition of experimental grower diets (fed from 14 to 31 d of age) containing soybean meal (SBM) sunflower seed meal (SFM) and dehulled rapeseed meal (dRSM) as-is or after additional toasting (tSBM, tSFM, tdRSM, respectively).

	SBM	tSBM	SFM	tSFM	dRSM	tdRSM
Ingredients, g/kg as-fed basis						
Soybean meal	200.0	-	-	-	-	-
Toasted Soybean meal	-	200.0	-	-	-	-
High protein Sunflower seed meal	-	-	200.0	-	-	-
Toasted HP Sunflower seed meal	-	-	-	200.0	-	-
Dehulled Rapeseed meal	-	-	-	-	200.0	-
Toasted dehulled Rapeseed meal	-	-	-	-	-	200.0
Wheat	250.0	250.0	191.2	191.2	183.6	183.6
Maize	298.1	298.1	347.3	347.3	347.3	347.3
Maize gluten (Prairy gold)	4.1	4.1	1.5	1.5	17.3	17.3
Finely ground oat hulls	29.7	29.7	-	-	-	-
Soy protein (Provisoy)	39.3	39.3	32.2	32.2	44.7	44.7
Peas	84.9	84.9	100.0	100.0	100.0	100.0
Potato protein	5.0	5.0	30.0	30.0	19.1	19.1
Soybean oil	20.6	20.6	29.8	29.8	23.6	23.6
Chicken fat	30.0	30.0	30.0	30.0	30.0	30.0
Limestone	9.5	9.5	9.1	9.1	7.3	7.3
Mono calcium phosphate	7.4	7.4	6.6	6.6	6.4	6.4
Sodium bicarbonate	3.5	3.5	4.3	4.3	3.9	3.9
Salt	0.7	0.7	-	-	0.3	0.3
DL-Methionine	2.6	2.6	1.7	1.7	1.9	1.9
L-Valine	0.1	0.1	-	-	-	-
L-Tryptophan	-	-	0.8	0.8	0.2	0.2
L-lysine HCl	2.4	2.4	4.7	4.7	3.5	3.5
L-Threonine	2.2	2.2	0.9	0.9	0.8	0.8
Phytase primix^1^	2.0	2.0	2.0	2.0	2.0	2.0
Premix^2^	5.0	5.0	5.0	5.0	5.0	5.0
TiO_2_^3^	1.0	1.0	1.0	1.0	1.0	1.0
Analyzed nutrients g/kg. DM (unless otherwise stated)						
DM, g/kg	889.6	893.4	895.1	898.0	891.7	892.2
Crude protein	234.2	233.9	238.4	237.0	238.7	238.3
Crude fat	64.1	80.0	85.6	90.4	80.2	85.2
Starch	436.1	431.4	428.0	420.6	428.0	420.6
Crude fiber	40.4	39.0	42.8	43.8	41.5	42.4
Soluble NSP (calculated)	29.0	29.0	26.8	26.8	29.1	29.1
Insoluble NSP (calculated)	116.6	116.6	119.7	119.7	117.8	117.8
Titanium	0.80	0.79	0.82	0.78	0.79	0.87
Metabolizable energy (MJ, calculated)	12.6	12.6	12.6	12.6	12.6	12.6

1 The phytase premix supplied 1000 FTU/kg (calculated).

2 Premix provided per kilogram of diet: 10000 IU vitamin A, 3300 IU vitamin D_3_, 50 mg vitamin E, 2.5 mg vitamin K_3_, 2.5 mg vitamin B_1_ (thiamine mononitrate), 7.5 mg vitamin B_2_ (riboflavin), 50 mg vitamin B_3_ (niacin), 15 mg pantothenic acid, 5 mg vitamin B_6_, 25 µg vitamin B_12_, 250 µg biotin, 500 mg choline chloride, 435 mg, 1.5 mg folic acid, 29 mg linoleic acid (C18:2), 100 mg Ca, 10.2 mg P, 6.8 mg Mg, 14.8 mg K, 0.6 mg Na, 111.3 mg Cl, 76.5 mg S, 57.2 mg Fe (as FeSO_4_), 2.0 mg I (as Ca(IO_3_)_2_), 12.5 mg Cu (as CuSO_4_), 75.4 mg Mn (MnO), 70.1 mg Zn (ZnSO_4_), 0.25 mg Se (as Na_2_SeO_3_), 0.8 mg butylated hydroxytoluene, 0.8 mg propyl gallate, 2.0 mg citric acid. Ground wheat was used as carrier.

3 Only diets fed from d 21 onwards contained TiO2, where it was exchanged for maize.

**Table 3:** Analyzed amino acid composition of experimental grower diets (fed from 14 to 31 d of age) containing soybean meal (SBM), dehulled sunflower seed meal (dSFM) and dehulled rapeseed meal (dRSM) as-is or after additional toasting (tSBM, tSFM, tdRSM, respectively).

	SBM	tSBM	dSFM	tdSFM	dRSM	tdRSM
Analyzed amino acids (AA), g/kg DM total diet						
*Essential amino acids*						
Arginine	14.5	14.3	15.5	15.4	13.9	14.6
Cysteine	4	3.9	4.1	4.1	5	5
Histidine	6.4	6.4	6.8	6.9	7	6.8
Isoleucine	9.5	9.6	9.5	9.7	9.4	9.5
Leucine	18.1	18.1	17.6	17.9	19.3	19.6
Lysine	13.5	13.4	14	13.7	14.7	14.8
Methionine	6.4	5.7	6.4	6.4	6.4	6.6
Threonine	10.3	10.4	9.7	9.6	9.9	10
Valine	10.6	10.6	11.2	11.4	11.4	11.5
*Nonessential amino acids*						
Alanine	10.5	10.5	10.5	10.9	11.4	11.5
Aspartate	21.3	21.7	20.3	20.4	18.5	18.6
Glutamate	44.1	44.3	43.3	43.6	43	43.6
Glycine	9.2	9.3	11.1	11.2	10.3	10.4
Proline	14.8	14.8	13.5	13.9	15.5	14.5
Serine	11.1	11.2	10.6	10.7	10.7	10.8

### Measurements

Individual body weight (**BW**) and feed and water intake per pen were recorded weekly. On d 23, litter quality was scored by an accessor blinded to the experimental treatments according to the method of Van Harn et al. (2009), in which a score was given from 1 to 10 for wetness (1 = very wet and 10 = completely dry) and for friability (1 = bedding has become one hard agglomeration and 10 = completely loose bedding). The scores for wetness and friability were averaged per pen.

At d 24, half of the birds were removed. The remaining 5 birds per pen were all males and had a BW closest to the pen average. The cardboard and bedding were removed from the pens three days prior to the dissection on d 29, 30, or 31, allowing the collection of excreta via the slatted floor. Excreta were collected cumulatively per pen once a day for 3 days and stored at −20°C pending analyses.

Dissections were performed on d 29 (3 blocks), d 30 (3 blocks) and d 31 (2 blocks). Birds were fasted from 6 to 3 h before euthanasia and subsequently allowed to consume feed until euthanasia, in order to ensure sufficient digesta in the ileum for analyses. Birds were euthanized by injection of sodium pentobarbital in the wing vein. Light was on during the night before dissections to ensure feed intake.

Body weight was determined immediately postmortem, and thereafter, the body cavity was opened and the GI-tract removed. The ileum (from Meckel's diverticulum to the ileocecal valve) was tied with tie-raps before separation, after which it was separated in 2 equal parts. The digesta of the distal half of the ileum was collected by flushing with demineralized water. Distal ileum digesta were pooled per pen, and stored at −20°C pending analyses.

### Analytical Procedures

Frozen excreta were partly thawed at room temperature for a maximum 6 h, pooled per pen, homogenized using the FGC 10-2 Feuma cutter (Feuma Gastromaschinen GmbH, Göβnitz, Germany) and subsequently freeze-dried. Pooled ileal digesta samples were freeze-dried.

Diets, freeze-dried ileal digesta and freeze-dried excreta samples were ground to pass a 1-mm screen using a Retsch ZM 100 mill prior to chemical analysis. Dry matter (**DM**; ISO 6496; ISO, 1999a), nitrogen (**N**; Dumas method; ISO 16634-1; ISO 2008) and Ti (Short et al., 1996) were determined in these samples. Uric acid was determined by enzymatic-colorimetry using a kit (Uric Acid liquicolor plus, 10694, Human GmbH, Wiesbaden, Germany). Furthermore, diets were analyzed for starch (ISO 15914; ISO, 2004), crude fat (after HCl hydrolysis; ISO 6492; ISO, 1999b) and crude fiber (ISO 6865; ISO, 2000).

Test ingredients, diets, freeze-dried ileal digesta and freeze-dried excreta samples were analyzed for AA. Sulfur-containing AA were analyzed after oxidation with performic acid (ISO 13903; ISO, 2005).

### Calculations and Statistical Analyses

Fecal N was calculated as total N in the excreta minus N from uric acid. Crude protein (**CP**) was calculated as N x 6.25. Apparent ileal digestibility (**AID**) or apparent total tract digestibility **(ATTD)** of DM, N and AAs were calculated according to the following equation:

$$\text{AID}\;\text{or}\;\text{ATTD}\;=\;\frac{{\left(\text{nutrient}/\text{Ti}\right)}_{\text{diet}}\;-\;{\left(\text{nutrient}/\text{Ti}\right)}_{\text{digesta}}}{{\left(\text{nutrient}/\text{Ti}\right)}_{\text{diet}}}\;\times \;100$$

in which, (nutrient/Ti)_diet_ represents the ratio of the nutrient of interest (DM, N or an AA) and Ti in the diet and (nutrient/Ti)_digesta_ represents this ratio of nutrient and Ti in the ileal digesta (for AID) or in feces (for ATTD). All concentrations are in g/kg.

Flows of N, DM and AA in ileal digesta or feces were calculated as:

$$\text{Flo}w_{\text{nutrient}}\;=\;\text{intak}e_{\text{ti}\;}\;\times \;\text{co}n_{\text{nutrient}}/\text{co}n_{\text{ti}}$$

in which, Flow_nutrient_ (g/day) is the amount of N, DM or AA passing the ileum or being excreted, intake_ti_ is the amount of Ti ingested (g/day), con_nutrient_ is the concentration of N, DM or the AA in the digesta or excreta (g/kg) and con_ti_ is the concentration of Ti in the digesta or excreta (g/kg). The fecal-ileal flow difference is calculated as fecal flow minus ileal flow.

All statistical analyses were performed using SAS (version 9.4, SAS Intitute Inc., Cary, NC). Performance data, litter quality scores, AIDs, ATTDs, and nutrient flows were analyzed by ANOVA using the PROC GLM procedure with the following statical model:

$$Y_{\text{ijk}}\;=\;\mu \;+\;P_{i}\;+\;T_{j}\;+\;P_{i}\;\times \;T_{j}\;+\;e_{\text{ijk}}$$

where Y_ijk_ represents the measured response of birds fed the ith protein source (i = SBM, SFM or dRSM) with the jth toasting level (j = as is or additionally toasted), housed in the kth pen (k =1–48), µ the overall mean, P_i_ the effect of the protein source tested, T_j_ the effect of toasting this protein source, P_i_ × T_j_ the interaction effect of protein source and toasting level and e_ijk_ the error. The corresponding room or block were included into the model as random factors when their effect was significant (*P* < 0.05). In case of a significant interaction effect or protein source effect, posthoc least square mean comparisons were Tukey adjusted.

Performance data were analyzed for 2 phases: from hatch to 14 d of age, the starter phase, and from 14 to 24 d of age, the grower phase. Initially, performance parameters were analyzed with percentage of females in a pen as a co-variable. Since this did not result in any significant effect, it was excluded from the final analysis. The sum of body weights per pen was included as a co-variable in the model for litter score.

## RESULTS

### Performance

Pen average initial body weight was 44.1 g (SE 0.1) per broiler and did not differ between treatments (protein source effect *P* = 0.19, toasting *P* = 0.62, interaction *P* = 0.77). Performance data are shown in Table 4. Additional toasting increased average daily feed intake (**ADFI**) and average daily gain (**ADG**), but only when feeding dRSM (interaction *P* < 0.001 for both), in the starter phase. Feed conversion ratio (**FCR**) was lower for the dRSM group without the additional toasting than all other groups (interaction *P =* 0.001) . Broilers fed SBM had higher ADFI and ADG than other sources in the starter phase (*P* < 0.01). In the grower phase, additional toasting reduced ADFI and ADG when feeding SFM, but not when feeding the other protein sources (interaction *P* < 0.001 and *P* = 0.01, respectively). Additional toasting did, however, increase the FCR of the dRSM fed birds (interaction *P* = 0.04). Furthermore, additional toasting reduced litter quality scores of broilers fed dRSM, but not of broilers fed SBM and SFM (interaction *P* = 0.04). ADFI and ADG were also affected by protein source, where SBM-fed birds had the highest intake and growth and the dRSM-fed birds the lowest (*P* < 0.001 for both). Litter quality scores negatively correlated with the sum of the sum of body weights in the pen (P = 0.006) and was therefore included as co-variable in that model.

**Table 4:** Growth performance of broilers fed diets containing soybean meal (SBM), dehulled sunflower seed meal (dSFM) or dehulled rapeseed meal (dRSM) as-is or after additional toasting (tSBM, tSFM, tdRSM, respectively).

	Dietary treatments							*P*-values		
	SBM	tSBM	SFM	tSFM	dRSM	tdRSM	pooled SEM	Protein source	Toasting	Protein source Toasting
Parameters^1^										
*Starter phase (0 to 14 d of age)*										
ADFI (g/d)	39.0^a^	37.3^ab^	35.7^bc^	33.8^cd^	28.4^e^	32.4^d^	0.61	**<0.001**	0.77	**<0.001**
ADG (g/d)	37.4^a^	35.9^ab^	34.7^bc^	32.4^cd^	28.3^e^	30.9^de^	0.70	**<0.001**	0.28	**<0.001**
FCR (g/g)	01.04^a^	1.03^a^	1.03^a^	1.05^a^	0.99^b^	1.05^a^	0.009	0.11	**0.004**	**0.001**
BW d14 (g)	568.0^a^	554.2^ab^	529.6^b^	497.6^c^	444.3^d^	475.7^c^	8.42	**<0.001**	0.40	**<0.001**
CV (%)	09.4	14.2	11.9	11.8	13.7	10.3	1.94	0.99	0.81	0.08
ADWI (ml/d)	96.2^a^	97.4^a^	96.2^a^	90.5^a^	80.7^b^	93.7^a^	2.87	**<0.001**	0.09	**<0.001**
WFR (ml/g)	2.64	2.62	2.70	2.69	2.86	2.91^c^	0.055	**<0.001**^3^	0.16	0.31
*Grower phase (14 to 24 d of age)*										
ADFI (g/d)	120.0^a^	118.8^ab^	113.2^b^	104.5^c^	93.9^cd^	99.6^d^	1.73	**<0.001**	0.31	**<0.001**
ADG (g/d)	89.5^a^	87.3^a^	85.5^a^	76.4^b^	71.2^b^	72.9^b^	1.58	**<0.001**	**0.02**	**0.01**
FCR (g/g)	001.34abc	001.36abc	001.33^ac^	001.37^ab^	1.31^c^	1.39^d^	0.011	0.97	**<0.001**	**0.04**
BW d24 (g)	1461^a^	1436^a^	1381^a^	1269^b^	1157^c^	1204^bc^	21.3	**<0.001**	0.08	**0.002**
CV (%)	10.2	13.1	12.1	18.7	12.6	14.0	1.61	0.06	**0.01**	0.23
ADWI (ml/d)	237.2^a^	232.8^ab^	245.3^a^	219.0^bc^	203.7^c^	219.2^bc^	4.52	**<0.001**	0.15	**<0.001**
WFR (ml/g)	1.98	1.96	2.13	2.10	2.18	2.20	0.039	**<0.001**^4^	0.80	0.78
*Litter quality*										
Score^2^	6.31^a^	6.06^a^	6.13^a^	6.06^ab^	6.56^a^	5.50^b^	0.26	0.74	**0.008**	**0.042**

a-e In case of a protein source × toasting interaction: means within a row lacking a common superscript letter differed significantly (*P* < 0.05).

1 ADFI: average daily feed intake, ADG: average daily gain, FCR: feed conversion ratio, BW: body weight on day 14 or 24, CV: coefficient of variation in individual gain, ADWI: average daily water intake, WFR: water to feed ratio.

2 Litter scores were given from 1 to 10 in which 1 = ‘very wet and agglomerated litter’ and 10 = ‘perfectly dry and loose litter’ at 23 d of age. The sum of BW of all birds in a pen was included as co-variable in the statistical model.

3 For WFR in the starter phase, all three protein sources differed from one and other.

4 For WFR in the grower phase, SBM differed from SFM and dRSM.

### Digestibility

Toasting reduced AID of DM (*P* = 0.04) and did not differ between protein sources (Table 5). The AID of N was reduced by toasting, but only when feeding dRSM (interaction *P* = 0.009). Toasting affected the AID of most AAs, but this depended on the protein source. The AID of cysteine, histidine, lysine, methionine, and aspartate were reduced when feeding toasted SBM, and the AID of cysteine, histidine, lysine, aspartate, glycine, and proline were reduced when feeding dRSM. Toasting SFM had no effect on AID of any of the measured AA. Furthermore, protein source affected the AID of some of the AAs, which generally indicated a higher AID when feeding SBM, except for arginine which was highest when feeding SFM.

**Table 5:** Apparent ileal digestibility coefficients^1^ of diets containing soybean meal (SBM), dehulled sunflower seed meal (dSFM) or dehulled rapeseed meal (dRSM) as-is or after additional toasting (tSBM, tSFM, tdRSM, respectively).

	Dietary treatments							*P*-values		
	SBM	tSBM	SFM	tSFM	dRSM	tdRSM	pooledSEM	Protein source	Toasting	Protein source × Toasting
Dry matter	71.5	69.6	69.4	69.7	70.7	66.8	1.06	0.25	**0.04**	0.17
Nitrogen	81.4^a^	78.6^a^	78.4^a^	78.4^a^	79.2^a^	74.4^b^	0.79	**<0.001**	**<0.001**	**0.01**
*Essential amino acids*										
Arginine	86.9	84.9	86.6	87.3	86.0	84.7	0.57	**0.04**^2^	0.07	0.07
Cysteine	74.1^a^	70.0^b^	72.4^ab^	72.8^ab^	75.2^a^	69.4^b^	0.85	0.84	**<0.001**	**0.004**
Histidine	83.2^ab^	80.0^c^	80.8^ac^	80.5^ac^	83.9^b^	78.5^c^	0.71	0.33	**<0.001**	**0.002**
Isoleucine	83.0^a^	80.9^a^	80.0^ab^	81.4^a^	80.4^ab^	77.3^b^	0.79	**0.002**	0.07	**0.02**
Leucine	84.3	82.3	81.8	83.0	82.1	80.1	0.77	**0.03**^3^	0.17	0.08
Lysine	87.6^a^	84.7^bc^	86.8^ab^	86.1abc	87.9^a^	83.6^c^	0.57	0.53	**<0.001**	**0.01**
Methionine	90.4^a^	87.6^b^	87.6^b^	87.9^b^	88.7^ab^	87.4^b^	0.59	0.05	**0.01**	**0.02**
Threonine	79.5	77.5	74.9	75.8	76.0	71.7	1.00	**<0.001**^4^	**0.04**	0.06
Valine	81.0^a^	78.9^ab^	78.3^ab^	79.5^ab^	79.5^ab^	76.2^b^	0.83	0.05	**0.04**	**0.02**
*Nonessential amino acids*										
Alanine	81.8^a^	79.2^ab^	79.2^ab^	80.6^ab^	80.8^ab^	77.7^b^	0.88	0.43	0.06	**0.04**
Aspartate	79.9^a^	75.7^bc^	77.5^ab^	75.9^bc^	78.9^ab^	72.5^c^	0.88	0.08	**<0.001**	**0.04**
Glutamate	88.4^a^	86.2^ab^	87.1^ab^	87.5^ab^	87.5^ab^	85.1^b^	0.57	0.22	**0.01**	**0.03**
Glycine	78.1^ab^	75.2^ac^	72.6^cd^	71.7^d^	78.4^b^	72.6^cd^	0.79	**<0.001**	**<0.001**	**0.01**
Proline	85.0^a^	83.2^a^	82.5^a^	83.7^a^	83.4^a^	77.9^b^	0.70	**<0.001**	**0.001**	**<0.001**
Serine	80.8^a^	78.7^ab^	75.9^ac^	77.1^ac^	77.3abc	73.8^c^	0.89	**<0.001**	0.06	**0.04**

a-d In case of a protein source × toasting interaction: means within a row lacking a common superscript letter differed significantly (*P* < 0.05).

1 Apparent ileal digestibility coefficient $=\frac{{\left(\text{nutrient}/\text{Ti}\;\text{marker}\right)}_{\text{diet}}\;-\;{\left(\text{nutrient}/\text{Ti}\;\text{marker}\right)}_{\text{ileum}\;\text{digesta}}}{{\left(\text{nutrient}/\text{Ti}\;\text{marker}\right)}_{\text{diet}}}\times \;100$.

2 For arginine, SFM differed from dRSM, but both did not differ from SBM.

3 For leucine, SBM differed from dRSM, but both did not differ from SFM.

4 For threonine, SBM differed from SFM and dRSM.

The ATTD of DM,N, and most AA was highest for SBM (Table 6). Additional toasting reduced ATTD of DM and N with the dRSM fed groups, but not for other protein sources (interaction *P* < 0.001 for both). Toasting generally reduced the ATTD of all AA, but this effect depended on the protein source. The ATTD of all measured AA reduced when toasting dRSM, while only histidine, lysine, aspartate, and proline reduced when toasting SBM. Toasting SFM only reduced the ATTD of lysine.

**Table 6:** Apparent total tract digestibility coefficients^1^ of diets containing soybean meal (SBM), dehulled sunflower seed meal (dSFM) or dehulled rapeseed meal (dRSM) as-is or after additional toasting (tSBM, tSFM, tdRSM, respectively).

	Dietary treatments							*P*-values		
	SBM	tSBM	SFM	tSFM	dRSM	tdRSM	pooledSEM	Protein source	Toasting	Protein source × Toasting
Dry matter	70.4^a^	70.2^a^	68.1^b^	68.1^b^	69.4^ab^	64.9^c^	0.44	**<0.001**	**<0.001**	**<0.001**
Nitrogen	77.6^a^	76.9^a^	71.8^b^	71.7^b^	76.4^a^	70.8^b^	0.51	**<0.001**	**<0.001**	**<0.001**
*Essential amino acids*										
Arginine	88.8^a^	87.8^a^	88.1^a^	87.7^a^	88.6^a^	86.2^b^	0.29	**0.011**	**<0.001**	**0.005**
Cysteine	74.3	72.3	71.5	70.6	71.7	69.0	0.54	**<0.001**^3^	**<0.001**	0.26
Histidine	83.3^a^	80.2^bc^	81.7^ab^	80.5^b^	84.0^a^	78.1^c^	0.54	0.33	**<0.001**	**<0.001**
Isoleucine	83.0^a^	81.3^ab^	80.4^b^	80.1^b^	82.3^a^	77.3^c^	0.41	**<0.001**	**<0.001**	**<0.001**
Leucine	84.3^a^	82.9^ab^	81.9^b^	81.4^bc^	84.1^a^	80.2^c^	0.39	**<0.001**	**<0.001**	**<0.001**
Lysine	87.0^a^	85.1^b^	84.6^b^	83.1^c^	87.6^a^	82.4^c^	0.26	**<0.001**	**<0.001**	**<0.001**
Methionine	88.8	87.3	87.5	87.6	88.3	86.5	0.43	0.30	**0.002**	0.06
Threonine	80.2^a^	79.0^a^	75.8^b^	74.8^b^	78.3^a^	72.6^c^	0.47	**<0.001**	**<0.001**	**<0.001**
Valine	80.6^ab^	79.1^bc^	78.5^c^	78.2^c^	81.2^a^	76.1^d^	0.44	**=0.004**	**<0.001**	**<0.001**
*Nonessential amino acids*										
Alanine	78.7^ab^	76.8^ac^	77.2^ac^	77.1^ac^	80.6^b^	75.7^c^	0.48	0.12	**<0.001**	**<0.001**
Aspartate	81.8^a^	78.9^b^	79.4abc	77.2^c^	81.3^ab^	74.1^d^	0.57	**<0.001**	**<0.001**	**<0.001**
Glutamate	88.4^a^	87.2^ab^	87.5^ab^	87.1^b^	88.1^ab^	84.9^c^	0.32	**0.001**	**<0.001**	**<0.001**
Glycine	66.1^a^	65.4^a^	66.0^a^	66.4^a^	71.8^b^	64.2^a^	0.96	0.06	**0.002**	**<0.001**
Proline	85.2^a^	83.5^bc^	82.4^c^	82.6^c^	84.4^ab^	77.9^d^	0.38	**<0.001**	**<0.001**	**<0.001**
Serine	81.5^a^	80.0^a^	77.0^b^	76.2^bc^	79.5^a^	74.1^c^	0.52	**<0.001**	**<0.001**	**<0.001**

a-d In case of a protein source × toasting interaction: means within a row lacking a common superscript letter differed significantly (*P* < 0.05).

1 Apparent total tract digestibility coefficient $=\frac{{\left(\text{nutrient}/\text{Ti}\;\text{marker}\right)}_{\text{diet}}\;-\;{\left(\text{nutrient}/\text{Ti}\;\text{marker}\right)}_{\text{excreta}}}{{\left(\text{nutrient}/\text{Ti}\;\text{marker}\right)}_{\text{diet}}}\times \;100$ ^2^Uric acid nitrogen measured in the excreta was excluded from the digestibility calculation.

3 For cysteine, SBM differed from SFM and dRSM.

### Nutrient flows through the intestinal tract

Feeding additionally toasted dRSM increased the ileal flow of DM and N, but had no effect within the SBM and SFM-fed birds (Table 7, interaction *P* = 0.003 and *P* = 0.001, respectively). The ileal flow of cysteine, histidine, lysine, threonine, valine, aspartate, glutamate, glycine, and proline when toasting dRSM, while only histidine, lysine and aspartate increased when SBM and none when toasting SFM. Daily fecal flows of DM and N decreased when toasting SFM, while it increased when toasting dRSM and was unaffected when toasting SBM (Table 8, interaction *P* < 0.001 for both). Fecal flows of all the measured AA increased when toasting dRSM, while only histidine, lysine and aspartate increased when toasting SBM. Toasting SFM had no effect on fecal AA flows. The fecal-ileal flow difference of N and lysine was highest for SFM fed birds (*P* < 0.001), but was not affected by toasting in any of the protein sources fed (Table 9). SBM fed birds had a lower, negative, fecal-ileal flow difference for cysteine (*P* = 0.006) and a higher, positive difference for glycine (*P* = 0.001).

**Table 7:** Ileal digesta nutrient flows^1^ in broilers fed diets containing soybean meal (SBM), dehulled sunflower seed meal (dSFM) or dehulled rapeseed meal (dRSM) as-is or after additional toasting (tSBM, tSFM, tdRSM, respectively).

	Dietary treatments							*P*-values		
	SBM	tSBM	SFM	tSFM	dRSM	tdRSM	pooledSEM	Protein source	Toasting	Protein source × Toasting
Dry matter (g/d)	030.4^a^	032.3^a^	031.0^a^	028.3^ab^	024.5^b^	029.3^a^	01.0	**<0.001**	0.13	**0.003**
Nitrogen	744.4^ab^	851.0^a^	832.7^a^	766.7^ab^	664.6^b^	861.7^a^	32.8	0.45	**0.01**	**0.001**
*Essential amino acids*										
Arginine	202.4^ab^	229.4^b^	210.5^ab^	183.7^a^	162.5^c^	197.5abc	09.3	**0.001**	0.14	**0.002**
Cysteine	110.6^ab^	124.5^ac^	113.7^ab^	103.6^b^	103.0^b^	133.8^a^	04.1	**0.03**	**0.001**	**<0.001**
Histidine	115.4^a^	136.4^b^	133.0^ab^	126.9^ab^	093.7^c^	130.3^ab^	05.4	**0.001**	**<0.001**	**0.001**
Isoleucine	172.5^ab^	194.8^b^	193.6^b^	168.3^ab^	154.8^a^	189.6^ab^	08.3	0.23	0.13	**0.001**
Leucine	303.5^a^	339.9^a^	324.8^a^	285.9^a^	289.8^a^	344.4^a^	15.1	0.50	0.18	**0.01**
Lysine	178.1^ab^	217.9^c^	187.9^ac^	179.8^ab^	148.7^b^	214.4^ac^	09.1	0.09	**<0.001**	**0.001**
Methionine	065.1^a^	075.2^ab^	080.6^b^	072.9^ab^	060.7^a^	072.7^ab^	04.1	**0.02**	0.14	**0.02**
Threonine	226.0^ab^	248.0^b^	246.8^b^	219.2^ab^	199.8^a^	250.2^b^	10.8	0.37	0.10	**0.003**
Valine	214.0^ab^	237.8^b^	247.1^b^	219.3^ab^	195.4^a^	241.8^b^	10.3	0.23	0.10	**0.002**
*Nonessential amino acids*										
Alanine	204.1^ab^	230.8^a^	221.5^ab^	199.0^ab^	182.4^b^	227.0^ab^	10.4	0.32	0.06	**0.01**
Aspartate	456.1^a^	558.8^b^	463.4^a^	462.5^a^	325.9^c^	452.2^a^	20.5	**<0.001**	**<0.001**	**0.01**
Glutamate	548.3abc	648.5^b^	567.4^ab^	511.3^ac^	449.4^ab^	572.9^c^	27.2	**0.003**	**0.01**	**0.002**
Glycine	214.2^ab^	244.3^b^	308.0^c^	297.3^c^	186.9^a^	250.8^b^	09.8	**<0.001**	**0.001**	**0.001**
Proline	237.2^ab^	264.6^b^	239.7^ab^	213.1^a^	216.2^a^	281.7^b^	11.1	**0.04**	**0.02**	**0.001**
Serine	228.0^ab^	253.0^b^	258.7^b^	230.3^ab^	203.6^a^	250.3^ab^	10.7	0.13	0.11	**0.003**

a-d Means within a row lacking a common superscript letter differ significantly (*P* < 0.05).

1 Ileal digesta nutrient flow = intake Ti (g/day) x concentration nutrient in digesta / concentration Ti in digesta. Flows are in mg/day unless stated otherwise.

**Table 8:** Fecal nutrient flows^1^ in broilers fed diets containing soybean meal (SBM), dehulled sunflower seed meal (dSFM) or dehulled rapeseed meal (dRSM) as-is or after additional toasting (tSBM, tSFM, tdRSM, respectively).

	Dietary treatments							*P*-values		
	SBM	tSBM	SFM	tSFM	dRSM	tdRSM	pooledSEM	Protein source	Toasting	Protein source × Toasting
Dry matter (g/d)	31.6^ab^	31.6^ab^	32.4^a^	29.8^b^	25.6^c^	31.2^ab^	0.5	**<0.001**	**0.012**	**<0.001**
Nitrogen^2^	885.4^a^	935.8^ab^	1094.0^c^	1023.9^bc^	761.0^d^	998.9^b^	21.9	**<0.001**	**<0.001**	**<0.001**
*Essential amino acids*										
Arginine	173.1^a^	185.7^a^	187.7^a^	177.1^a^	132.1^b^	178.8^a^	4.5	**<0.001**	**<0.001**	**<0.001**
Cysteine	109.9^a^	115.3^a^	117.3^a^	111.8^a^	117.3^a^	136.2^b^	3.0	**<0.001**	**0.01**	**0.001**
Histidine	114.7^a^	134.7^b^	126.5^ab^	126.8^ab^	093.3^c^	133.4^b^	4.2	**0.002**	**<0.001**	**<0.001**
Isoleucine	173.2^a^	190.5^a^	189.4^a^	179.8^a^	139.1^b^	191.0^a^	4.4	**<0.001**	**<0.001**	**<0.001**
Leucine	304.3^a^	328.1^ab^	322.9^ab^	312.1^a^	256.3^c^	345.4^b^	7.8	0.07	**<0.001**	**<0.001**
Lysine	186.0^b^	212.5^c^	218.7^cd^	216.9^cd^	152.7^a^	230.3^d^	4.5	**<0.001**	**<0.001**	**<0.001**
Methionine	076.3^a^	077.2^a^	080.8^a^	074.5^a^	062.3^b^	078.4^a^	2.8	**0.01**	0.09	**<0.001**
Threonine	218.6^a^	232.2^ab^	237.6^ab^	227.3^ab^	180.3^c^	243.6^b^	5.4	**0.001**	**<0.001**	**<0.001**
Valine	218.6^a^	235.4^ab^	244.0^b^	232.7^ab^	178.4^c^	244.2^b^	5.5	**<0.001**	**<0.001**	**<0.001**
*Nonessential amino acids*										
Alanine	239.3^ab^	256.8^b^	242.7^ab^	233.7^a^	184.3^c^	248.8^ab^	5.5	**<0.001**	**<0.001**	**<0.001**
Aspartate	413.0^a^	484.8^b^	424.0^a^	435.4^ab^	288.4^c^	428.8^a^	12.1	**<0.001**	**<0.001**	**<0.001**
Glutamate	545.4^ab^	602.7^b^	546.8^a^	527.2^ab^	428.9^c^	584.6^ab^	14.4	**<0.001**	**<0.001**	**<0.001**
Glycine	332.5^a^	340.4^ab^	381.2^b^	352.2^ab^	243.0^c^	329.3^a^	11.3	**<0.001**	**0.012**	**<0.001**
Proline	234.1^bc^	259.3^ab^	240.6^bc^	225.7^cd^	202.4^d^	284.2^a^	6.2	0.09	**<0.001**	**<0.001**
Serine	220.0^a^	237.2^ab^	247.1^b^	238.5^ab^	183.6^c^	248.6^b^	6.1	**<0.001**	**<0.001**	**<0.001**

a-d Means within a row lacking a common superscript letter differ significantly (*P* < 0.05).

1 Fecal nutrient flow = intake Ti (g/day) x ‘concentration nutrient in feces’/‘concentration Ti in feces.’ Flows are in mg/day unless stated otherwise.

2 Values for nitrogen exclude nitrogen from uric acid.

**Table 9:** Fecal-ileal flow differences^1^ in broilers fed diets containing soybean meal (SBM), dehulled sunflower seed meal (dSFM) or dehulled rapeseed meal (dRSM) as-is or after additional toasting (tSBM, tSFM, tdRSM, respectively).

	Dietary treatments							*P*-values		
	SBM	tSBM	SFM	tSFM	dRSM	tdRSM	pooledSEM	Protein source	Toasting	Protein source × Toasting
Dry matter (g/d)	1.2	−0.7	1.4	1.5	1.0	1.8	1.0	0.33	0.73	0.33
Nitrogen^2^	141.0	84.8	261.3	257.1	96.4	132.4	33.1	**<0.001**^3^	0.74	0.40
*Essential amino acids*										
Arginine	−29.3	−43.7	−22.8	−6.6	−30.4	−18.6	8.94	0.06	0.56	0.19
Cysteine	−0.7	−9.2	3.6	8.2	14.3	2.3	4.22	**0.006**^4^	0.14	0.13
Histidine	−0.7	−1.7	−6.6	−0.1	−0.4	3.5	5.60	0.96	0.50	0.80
Isoleucine	0.7	−4.3	−4.2	11.5	−15.7	0.7	7.99	0.36	0.18	0.33
Leucine	0.8	−11.8	−1.9	26.2	−33.4	−0.8	14.70	0.14	0.20	0.25
Lysine	7.9	−5.5	30.8	37.1	4.1	15.6	7.84	**<0.001**^5^	0.82	0.26
Methionine	11.2	2.1	0.2	1.6	1.6	4.7	4.15	0.26	0.70	0.15
Threonine	−7.4	−15.8	−9.2	8.0	−19.5	−6.2	10.37	0.43	0.40	0.42
Valine	4.6	−2.5	−3.0	13.4	−17.0	1.7	9.76	0.43	0.25	0.36
*Nonessential amino acids*										
Alanine	35.2	26.0	21.2	34.7	2.0	21.4	9.72	0.11	0.35	0.31
Aspartate	−43.1	−74.0	−39.5	−27.0	−37.5	−20.4	20.83	0.33	0.96	0.45
Glutamate	−2.9	−45.8	−20.6	15.9	−20.5	9.7	27.25	0.70	0.74	0.28
Glycine	118.3	96.0	73.2	54.9	56.1	80.1	12.16	**0.001**^6^	0.54	0.13
Proline	−3.2	−5.3	0.9	12.7	−13.8	0.5	11.39	0.45	0.41	0.75
Serine	−8.0	−15.8	−11.6	8.2	−20.0	0.5	10.11	0.56	0.21	0.29

1 Fecal-ileal flow difference is fecal nutrient flow minus ileal nutrient flow. Nutrient flow = intake Ti (g/day) x ‘concentration nutrient in digesta or feces’/‘concentration Ti in digesta or feces.’ Values are in mg/day unless stated otherwise. Negative values indicate a net disappearance and positive values indicate a net gain of nutrient in the hind-gut.

2 Values for nitrogen in feces exclude nitrogen from uric acid.

3 For nitrogen, SFM differed from dRSM and SBM.

4 For cysteine, SBM differed from SFM and dRSM.

5 For lysine, SFM differed from dRSM and SBM.

6 For glycine, SBM differed from SFM and dRSM.

## DISCUSSION

The objective of this study was to evaluate the effects of additional toasting of SBM, SFM, and dRSM on the N and AA flows into the hind-gut of broilers, in order to find a model for studying effects of PF. An increase of these flows would be favorable in this respect. Toasting could be a suitable method to create this contrast within the same protein source, while limiting confounding effects associated with other methods of creating a contrast in PF.

### Growth Performance

An increase in protein digesta flows was expected to negatively impact growth performance (De Lange et al., 2003; Bryan et al., 2019). Indeed, toasting increased FCR, but only when feeding dRSM. In line with this, the largest contrasts in N and AA flows were found between dRSM and tdRSM. Intake of digestible AA was actually higher for broilers fed toasted dRSM than for broilers fed non-toasted dRSM. Therefore, this negative impact of toasting dRSM on feed efficiency is likely due to protein fermentation.

Interestingly, broilers fed dRSM without additional toasting had the lowest FCR in this study. Generally, SBM is a highly digestible protein source, with subsequent low FCRs when feeding SBM to broilers (Qaisrani et al., 2014). However, the dRSM used in the current study was produced from dehulled rapeseed. Dehulling provided a RSM product with a higher CP and lower fiber level compared to commercially available RSM, which we also expected to result in a higher protein digestibility. This higher protein digestibility of the nontoasted dRSM allowed us to create a large contrast in ileal N digestibility and flow with toasting in line with the objective of our study. In addition, the low feed intake of the dRSM fed birds could have played a role in the low FCR. A reduction in feed intake in broilers might improve nutrient utilization (as reviewed by Aftab et al., 2018). Finally, it is also important to note that over half of the crude protein in the diets (55%, 57%, and 61% for the SBM, SFM and dRSM, respectively) derived from other protein sources such as maize, maize gluten, soy protein, peas and potato protein. The slightly higher inclusion of these other protein sources in the dRSM diet might contributed to the relatively low FCR. The formulations of the normal and toasted diets within each protein source were equal, hence, effects of toasting within a protein source can be fully attributed to the effect of toasting.

### Protein Digestibility and Digesta Flows

Ileal protein digesta flows depend on feed intake and digestibility. A small portion of this protein flow consists of endogenous secretions and microbes, as 16 to 17 g AA /kg of DM intake are endogenous losses found in the distal ileum (Ravindran et al., 2004). These endogenous losses may be influenced by fiber and antinutritional factors (see for review Ravindran, 2016), and hence, could be affected by protein source and additional toasting. However, we cannot separate endogenous and dietary protein in the current data.

The effect of toasting on digestibility differed between protein sources. Toasting strongly reduced AID and ATTD of N and AA in the dRSM groups, had no effect in the SFM groups and only reduced digestibility of a few AA in the SBM groups. Reduced digestibility resulted in increased prececal protein digesta flows.

A reduction in digestibility due to the additional toasting was expected. Although initial heating of soybeans during the production of SBM improves digestibility (Burnet and Arnold, 1952; Hancock et al., 1990; Tousi-Mojarrad et al., 2014), severe processing reduces it (Messerschmidt et al., 2012). The SBM used was a commercial product, which had already been heat-treated. We applied additional toasting, which reduced the digestibility of some AA, similar to results observed by González-Vega et al. (2011) in pigs. Similar to these authors, we observed a darker color of the toasted SBM (data not shown), indicating Maillard reactions took place.

Toasting SFM had no effect on N and AA flows or digestibility in broilers in the current trial, despite that here we observed a darker color of the toasted meal as well. This is in contradiction with other research, in which hydrothermal processing of SFM reduced protein digestibility in pigs (Almeida et al., 2014) and in cecectomized cockerels (Zhang and Parsons, 1994). It could be that the toasting of SFM in the current study was too mild to have caused similar effects as observed by these authors, as the durations of hydrothermal processing in these aforementioned studies was 10 to 50 min longer than in the current study. Methods were also slightly different, in both studies SFM was autoclaved at 130°C (Almeida et al., 2014) or 121°C (Zhang and Parsons, 1994).

Despite this, toasting SFM did reduce feed intake and growth, and tended to increased FCR in broilers. Indicating that the loss in bioavailability of AA due to toasting is not fully reflected in loss in digestibility. This is the case for lysine as shown in a number of poultry and pig studies (Van Barneveld et al., 1994; Fernandez and Parsons, 1996; Rérat et al., 2002; Hulshof et al., 2017). Analyzed lysine may include early Maillard reaction products (Rutherfurd et al., 1997) which may be absorbed in the small intestine (Moughan et al., 1996), but are not always utilized post absorption (Hulshof et al., 2017). Reactive lysine (determined as O-methylisourea-reactive lysine) is considered a good indicator for availability of lysine (Rutherfurd et al., 1997). Indeed, in earlier work in our lab, toasting SFM for 30 min at 136°C reduced the percentage of reactive lysine from 90 to 73% (unpublished data).

Toasting dRSM, darkened the meal color, reduced digestibility and increased ileal and fecal flows of N and AA. This is in line with earlier research in which toasting rapeseed meal reduced protein digestibility in pigs (Salazar-Villanea et al., 2018) and reduced growth performance in broilers (Newkirk and Classen, 2002). The fact that broilers fed the tdRSM also had a numerically higher feed intake compared with dRSM contributed to this contrast in flow. These increased flows likely caused the higher FCR seen in these birds. As it is unlikely this increased FCR was caused by a reduced uptake of AA, because the birds in the tdRSM group ingested more ileal digestible AA, due to the higher feed intake. Also, the poorer litter quality observed in broilers fed tdRSM could be due to the increased flow of N and AA. Increased excreta moisture levels were observed when feeding broilers higher levels of CP (Namroud et al., 2008; Bench et al., 2016) or when feeding low digestible protein (Hossain et al., 2013). Both feeding strategies could lead to increased N and AA flows into the hind-gut, similar to the current study.

The difference between ileal and fecal AA flow, measured in the current study, is a net difference, and expected to be mainly due to microbial activity, as AA absorption from the ceca is low in adequately fed broilers (Karasawa and Maeda, 1994). Our data show that fecal-ileal flow difference of N and some AA is mostly influenced by protein source and not by additional toasting. SFM fed birds showed the highest net increase of N and lysine from the ileum to feces, which could be the result of microbial growth. Lysine can be synthesized by the microbiome, as demonstrated in pigs (Torrallardona et al., 2003). Moreover, lysine is a moderate constituent of pig ileum bacteria in comparison to other AA (Dai et al., 2010). However, the latter study also demonstrated that lysine is rapidly fermented by microbes. This we expect would result in a lower or negative fecal-ileal flow difference for lysine. Hence a net increase of lysine in the hind-gut is unlikely an indicator for bacterial growth. Furthermore, host endogenous excretions in the ceca could contribute to this net increase of lysine as well. Quantitative contributions of endogenous excretions in ceca are unknown.

Toasting did increase ileal N or AA flows in broilers fed SBM and dRSM, however, this did not increase the fecal-ileal flow differences of N or AA. Therefore, it remains unclear whether toasting also changed microbial activity in the hind-gut of broilers.

## Conclusion

The effect of additional toasting depended on the protein source, where ileal N and AA flows were mainly increased when feeding dRSM, which also reduced feed efficiency. Therefore of the three protein sources studied in this experiment, additional toasted dRSM appears to be the most suitable protein source to use in a model for inducing protein fermentation.
